# Identification of *Trypanosoma brucei* RMI1/BLAP75 Homologue and Its Roles in Antigenic Variation

**DOI:** 10.1371/journal.pone.0025313

**Published:** 2011-09-28

**Authors:** Hee-Sook Kim, George A. M. Cross

**Affiliations:** Laboratory of Molecular Parasitology, The Rockefeller University, New York, New York, United States of America; The University of Maryland, United States of America

## Abstract

At any time, each cell of the protozoan parasite *Trypanosoma brucei* expresses a single species of its major antigenic protein, the variant surface glycoprotein (VSG), from a repertoire of >2,000 *VSG* genes and pseudogenes. The potential to express different *VSG*s by transcription and recombination allows the parasite to escape the antibody-mediated host immune response, a mechanism known as antigenic variation. The active *VSG* is transcribed from a sub-telomeric polycistronic unit called the expression site (ES), whose promoter is 40–60 kb upstream of the *VSG*. While the mechanisms that initiate recombination remain unclear, the resolution phase of these reactions results in the recombinational replacement of the expressed *VSG* with a donor from one of three distinct chromosomal locations; sub-telomeric loci on the 11 essential chromosomes, on minichromosomes, or at telomere-distal loci. Depending on the type of recombinational replacement (single or double crossover, duplicative gene conversion, etc), several DNA-repair pathways have been thought to play a role. Here we show that *VSG* recombination relies on at least two distinct DNA-repair pathways, one of which requires RMI1-TOPO3α to suppress recombination and one that is dependent on RAD51 and RMI1. These genetic interactions suggest that both *RAD51*-dependent and *RAD51*-independent recombination pathways operate in antigenic switching and that trypanosomes differentially utilize recombination factors for *VSG* switching, depending on currently unknown parameters within the ES.

## Introduction

Monoallelic expression of multigene families occurs in a variety of cellular processes, including mating-type switching in yeasts, immunoglobulin gene diversification in B-cell development, odorant receptors, and surface antigen variation in several pathogens [1]-[7]. *Trypanosoma brucei* is a protozoan pathogen that causes African sleeping sickness. Only one allele of the variant surface glycoprotein (*VSG*) is expressed at a time in each trypanosome, yet the genome may contain more than 2,000 *VSG* genes and pseudogenes. About 15 sub-telomeric polycistronic expression sites (ES) contain a *VSG* ∼ 50 kb downstream of their promoters [8]-[10] and about 1 kb upstream of the telomeric repeat array. Only one ES can transcribe a *VSG* at any time and the rest are transcriptionally silent. Most *VSG*s are not associated with an ES (minichromosomal and ‘telomere-distal’, also referred to as ‘chromosome-internal’), and they lack promoters. Antigenic switching is caused mainly by switching the expressed *VSG* through DNA recombination. Infrequently, a new VSG can be activated by switching the transcriptional status among the active and silent ES (reviewed in [4], [11], [12]). Recombination-mediated *VSG* switching occurs preferentially by gene conversion (GC) rather than crossover [13]-[16], despite the fact that there are no clear advantages in switching through GC *in vivo*.

One of the major factors that control mitotic crossover is the RTR complex (also known as the BTB complex). This complex consists of a **R**ecQ-family helicase (**B**LM in mammals and SGS1 in budding yeast), a **T**opoisomerase III α, and **R**MI1/2 (**B**LAP75/18 in mammals and RMI1 in budding yeast). RMI is the third component that has been recently identified in several organisms [17]-[23]. The functions of the RTR complex have been documented extensively in yeasts and mammalian systems [21], [22], [24]-[31]. The complex safeguards genome integrity by influencing various aspects, including DNA replication, mitotic and meiotic recombination, and telomere dynamics. One of major functions of the RTR complex is to remove recombination intermediates that may accumulate during these processes. Genetic studies from budding yeast showed that the growth defect of *top3* is caused by the accumulation of recombination intermediates and the defect could be relieved by mutations in *SGS1* or in the *RAD51*-pathway [28], [32], [33]. Recombination intermediates accumulated when TOP3 enzyme activity was compromised [34]. A Holliday Junction (HJ) is structurally similar to a recombination intermediate, and the RTR complex is required for the removal of both structures. *In vitro* studies showed that human RMI1 (hRMI1) stimulates the double Holliday Junction (dHJ) dissolution activity of TOPO3α and the dHJ unwinding activity of BLM-TOPO3α [35], [36]. In yeast, RMI1 stimulates the ssDNA binding and relaxation activity of TOP3 *in vitro* [37]. hRMI1 interaction with TOPO3α appears to control TOPO3α enzyme activity, as hRMI1 mutants defective for interaction with TOPO3α, not for DNA, lose the ability to stimulate the dHJ dissolution activity of TOPO3α *in vitro* [38].

We showed previously that trypanosomes lacking TOPO3α significantly increased *VSG* switching and this increase was largely due to the elevated levels of *VSG* GC and *VSG* crossover [39]. The data suggested that the RTR complex is probably conserved in *T. brucei* and may play roles in *VSG* switching by controlling undesirable recombination intermediates arising between the active *VSG* and silent *VSG* donors. Here we identify the *T. brucei* RMI1 homologue and demonstrate that TbRMI1 interacts with TbTOPO3α and promotes productive *VSG* switching. We show that, similarly to the previously described TOPO3α defect, RMI1 deficiency increases *VSG* switching rate by promoting *VSG* gene conversion and crossover. Genetic interactions between *TbRMI1* and *TbRAD51* further reveal that antigenic variation is under control of multiple recombination pathways, depending on where the recombination occurs, and that *VSG* GC and ES GC are likely to be initiated by different triggers.

## Results

### Deletion of Tb927.3.1830, a *T. brucei* DUF1767 domain-containing protein, causes a growth defect

We demonstrated previously that *T. brucei* TOPO3α plays critical roles in recombination-mediated *VSG* switching [39]. RMI1 homologues that work together with TOPO3α-BLM (TOP3-SGS1 in budding yeast) have recently been identified in several model organisms [18]-[20], [23], [30]. RMI1 homologues share a signature domain at the N-terminus, called DUF1767, whose function is unknown. By BLAST searches, we found two *T. brucei* proteins that contain a DUF1767 domain at the N-terminus, Tb927.3.1830 and Tb927.8.2040.

**Figure 1:**
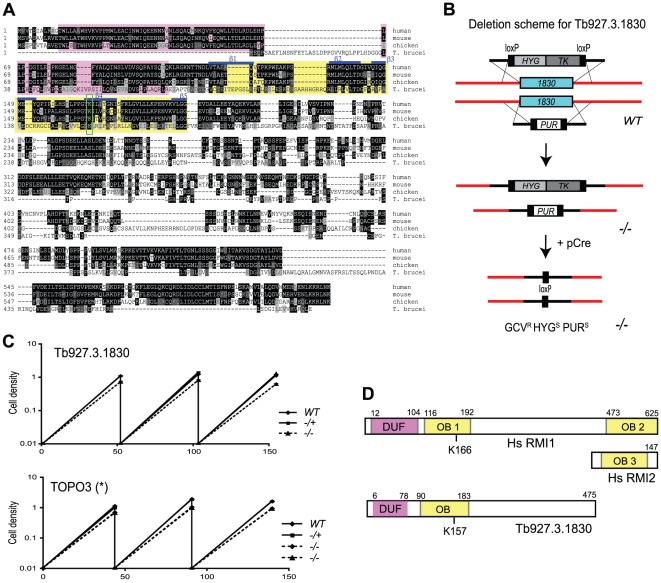
Deletion of Tb927.3.1830 causes a growth defect. (A) Alignment of Tb927.3.1830 with human, mouse and chicken RMI1. DUF1767 and OB-fold domains are indicated in pink and yellow boxes, respectively. Conserved lysine residues are in green box in the OB1 domain. Blue bars show locations of five β-strands of OB1. (B) Deletion scheme for Tb927.3.1830. The entire open reading frames (ORFs) of Tb927.3.1830 were sequentially deleted using cassettes containing *HYG-TK* or *PUR* flanked by loxP sites. The markers were removed by transient expression of Cre-recombinase [39], [64]. (C) *Tbrmi1* exhibits a minor growth defect, similar to *Tbtopo3α*. Wild-type, *rmi1^-/+^* and *rmi1^-/-^* cells were diluted to 10,000 cells/ml and cells were counted after two days of incubation. This was repeated twice. Growth phenotypes of *Tbtopo3α* mutants were taken from the previous study [39]. Error bars are shown, but are small. (D) Diagrams of human RMI1/2 [41] and Tb927.3.1830.

Figure 1A shows sequence alignment of Tb927.3.1830 with human, mouse, and chicken RMI1 homologues. RMI1 proteins possess an ‘oligonucleotide and oligosaccharide binding’ (OB)-fold (yellow box) next to the DUF1767 domain (pink box). In higher eukaryotes, RMI1 contains another OB-fold domain (OB2) in the C-terminus and associates with RMI2 (BLAP18) via the interaction between OB2 of RMI1 and OB3 of RMI2 [21],[22]. RMI1 deletion mimics TOP3 or TOPO3α deficiency in other organisms. Yeast *rmi1* mutant showed growth defects, similar to *top3* mutants [19], [23]. *Tbtopo3α* exhibited a minor growth defect [39]. To determine whether trypanosomes lacking any of these potential RMI1 homologues phenocopy *Tbtopo3α*'s growth defect, we constructed mutants lacking Tb927.3.1830 (Figure 1B) or Tb927.8.2040. Wild type, heterozygous and homozygous mutants were examined for cell growth. Deletion of Tb927.3.1830 mimicked *Tbtopo3α*, exhibiting a minor growth defect (Figure 1C), while Tb927.8.2040 deletion grew normally (data not shown), indicating that Tb927.3.1830 could be a RMI1 homologue but Tb927.8.2040 is probably not. Similar to *Tbtopo3α* mutant [39], Tb927.3.1830 deletion shows sensitivity to hydroxyurea, a drug that blocks replication (data not shown).

Human RMI core complex contains three OB domains, N-terminal OB1 and C-terminal OB2 in RMI1, and OB3 in RMI2 (Figure 1D). The OB1 of hRMI1 interacts with TOPO3α-BLM to form the BTB complex and the OB2 with OB3 of RMI2 to form the RMI subcomplex [22], [40]. X-ray crystal structure studies have shown that the RMI core complex interaction is essential for its *in vivo* function [40], [41]. The OB1 domain is essential for stimulation of the dHJ dissolution activity of the BLM-TOPO3α [40]. K166 (green box in Figure 1A) appears to be important for the stability of OB1 structure of hRMI1 [40]. *rmi1-K166A* mutant is unable to interact with TOPO3α and defective in stimulating the dissolution activity of BLM-TOPO3α [38]. This lysine residue seems to be conserved in *T. brucei* Tb927.3.1830 (K157 in green box in Figure 1A). But Tb927.3.1830 appears to lack OB2 domain and it is unknown whether there are multiple RMI proteins present in *T. brucei*.

### Tb927.3.1830 interacts with TbTOPO3α

**Figure 2:**
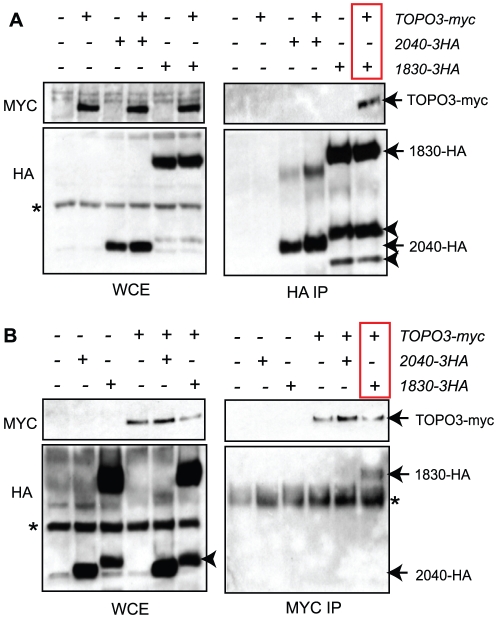
Tb927.3.1830 interacts with TbTOPO3α. (A) Tb927.3.1830-HA co-immunoprecipitates with TOPO3α-MYC. (B) TOPO3α-MYC co-immunoprecipitates with Tb927.3.1830-HA. TOPO3α was endogenously tagged with 3xMYC, and Tb927.8.2040 and Tb927.3.1830 with 3xHA. Cell lysates were immunoprecipitated either with anti-HA or anti-MYC antibodies and analyzed by western blot. (*) and (**) indicate antibody heavy and light chains. Arrow heads indicate break-down products of 1830-HA.

To confirm whether Tb927.3.1830 is a *T. brucei* RMI1 homologue, we performed co-immunoprecipitation experiments in cells expressing endogenous copies of Tb927.3.1830-3xHA or Tb927.8.2040-3xHA, and/or TOPO3α-3xMYC, each tagged at the C-terminus, using a PCR method [42]. The functionalities of TOPO3α-3xMYC and Tb927.3.1830-3xHA were confirmed by complementation of growth defects (data not shown). The functionality of Tb927.8.2040-3xHA could not be assessed as the mutant showed no phenotype but the tagging was made in a Tb927.8.2040 heterozygote and these grew normally. Lysates were prepared and immunoprecipitated with either anti-HA or anti-MYC antibodies. Precipitated proteins were analyzed by western blot. TOPO3α-3xMYC was pulled down only in Tb927.3.1830-3xHA immunoprecipitates, but not in Tb927.8.2040-3xHA (Figure 2A, red box). Consistent with this, TOPO3α-MYC coimmunoprecipitated with Tb927.3.1830, but not Tb927.8.2040 (Figure 2B, red box). These data confirm that Tb927.3.1830 is a RMI1 homologue, so Tb927.3.1830 will be referred to as TbRMI1 henceforth.

### TbRMI1 deficiency increases *VSG* switching frequency

**Figure 3:**
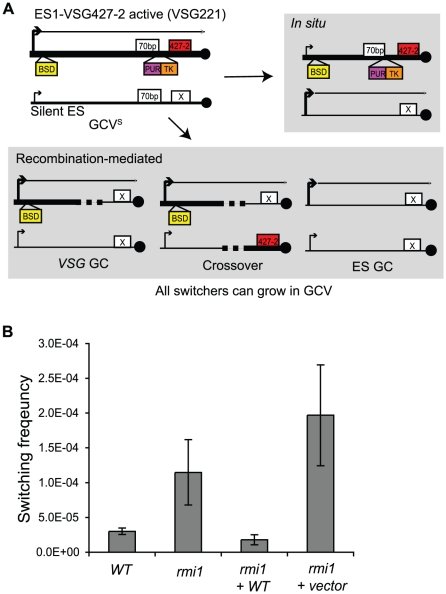
TbRMI1 deficiency increases *VSG* switching frequency. (A) *VSG* switching reporter cell line and switching mechanisms. The active expression site, ES1-*VSG* 427-2, was doubly marked with *BSD* at the promoter and *PUR-TK* immediately at the 3′ end of the 70-bp repeats. *VSG* switching events will accompany either loss or repression of the *PUR-TK* marker, which allows only the switchers to grow in the presence of GCV [39]. Non-switchers can be eliminated as described in the text. Black circles are telomere repeats. (B) TbRMI1 deficiency increases *VSG* switching frequency. Switching frequency was measured in wild type, *rmi1*, and *rmi1* cells transfected either with an empty vector or the wild-type *RMI1*.

Deletion of *TbTOPO3α* caused significant increase (10–40-fold) in *VSG* switching frequency [39]. Switching frequency should increase in the absence of TbRMI1, if TbRMI1 works together with TbTOPO3α in antigenic switching. We measured the *VSG* switching frequency in *Tbrmi1* null mutant, using the assay developed previously [39]. The active expression site, ES1-*VSG* 427-2 (*VSG* 221), was doubly marked with a blasticidin-resistance gene (*BSD*) and a *PUR-TK*, immediately downstream of the promoter and at the 3′ end of the 70-bp repeats, respectively (Figure 3A). *VSG* switching concurs with the loss or transcriptional repression of *PUR-TK* marker, so switched variants can be counter-selected in media containing ganciclovir (GCV), a nucleoside analogue that kills *TK*-expressing cells.

Wild type and *rmi1* strains were grown in media containing blasticidin and puromycin to maintain the homogeneity of the expressed *VSG*. The cells were then allowed to switch in the absence of selection for 3 days. 5×10^5^ or 1×10^6^ cells were suspended in media containing 5 µg/ml GCV and distributed in 96-well plates. Genuine switchers will be sensitive to puromycin. GCV-resistant clones that were not switched but carried mutations in *TK* were ruled out by examining puromycin sensitivity. *GCV^R^ PUR^S^* clones that were not switched and carried mutation(s) in *PUR-TK* should still express *VSG* 427-2. These were excluded by western blot using antibody against VSG 427-2. Five and three independent cultures from wild-type and *rmi1* mutants, respectively, were examined and the ratio of *GCV^R^* switchers to the total number of cells plated was plotted in Figure 3B. The results are also summarized in Table 1. Switching frequency increased ∼ 4 fold in *rmi1* (11.0±3.4×10^−5^), compared to wild type (2.8±0.5×10^−5^). To confirm that the switching phenotype was caused by the absence of *RMI1*, an empty vector or a vector containing wild type *RMI1* were introduced into the *rmi1* null mutant, and three independent clones from each transfection were examined for *VSG* switching frequency. As shown in Figure 3B, reintroduction of wild-type *RMI1* complemented the increased-switching phenotype of *rmi1* (1.8±0.7×10^−5^), while empty-vector transfected *rmi1* cells still switched at a higher frequency (20.0±7.2×10^−5^), indicating that increased-switching phenotype was due to RMI1 deficiency.

**Table 1:** *VSG*-switching events in wild type, *rmi1*, *rad51*, *rmi1 rad51*, and *rmi1 topo3α* cultures.

Genotype	Total # cells plated	Number of switchers					
		Total	*VSG* GC	XO	‘ES GC or loss’	*In situ*	ES+XO
*WT*	500,000	17	3	3	11	0	0
*WT*	500,000	11	5	0	6	0	0
*WT*	1,000,000	26	4	0	20	1	1
*WT*	1,000,000	26	9	0	16	1	0
*WT*	1,000,000	31	6	2	23	0	0
*rmi1*	500,000	74	56	16	2	0	0
*rmi1*	478,000	39	29	10	0	0	0
*rmi1*	500,000	50	34	14	2	0	0
*rad51*	2,000,000	39	30	4	2	3	0
*rad51*	2,000,000	26	9	4	1	9	3
*rad51*	2,000,000	10	7	1	1	0	1
*rmi1 rad51*	2,000,000	4	0	2	1	1	0
*rmi1 rad51*	2,000,000	2	0	1	0	1	0
*rmi1 rad51*	2,000,000	3	2	1	0	0	0
*rmi1 topo3α*	500,000	28	22	6	0	0	0
*rmi1 topo3α*	500,000	37	21	13	2	0	1

### TbRMI1 monitors *VSG* recombination, similarly to TbTOPO3α

**Figure 4:**
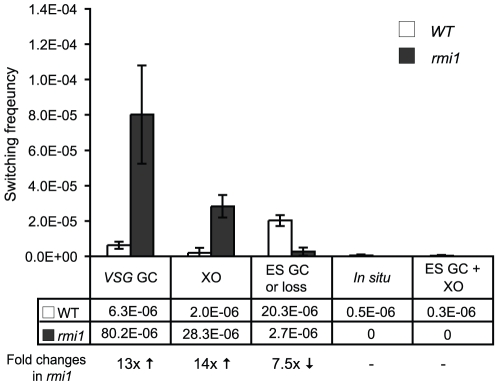
TbRMI1 monitors *VSG* recombination. TbRMI1 deficiency increases *VSG* GC and crossover (XO) but decreases ES GC. 275 cloned switchers were examined and the frequency of each switching mechanism was plotted. White bars are wild type and dark grey bars are *rmi1* mutant. Error bars indicate standard deviation. The results are also summarized in Table 1. Switched variants were assigned as follows (Figure 3A diagram); *VSG*-GC switchers, *BSD^+^ VSG* 427-2*^-^*; ‘ES GC or ES loss’ switchers, *BSD^-^ VSG* 427-2*^-^*; crossover switchers, *BSD^+^ VSG* 427-2*^+^*; *in situ*, *BSD^+^ VSG* 427-2*^+^*.

Hyper-recombination and elevated crossover are prominent phenotypes caused by TOPO3α deficiency in *T. brucei* [39] and in other organisms [26], [30], [31], [43], [44]. To determine whether TbRMI1 is required to suppress *VSG* GC and crossover, 275 cloned switchers (112 from five wild type cultures and 163 from three *rmi1* cultures) were examined. The diagram in Figure 3A illustrates four major switching mechanisms characterized. Each event can be distinguished by assessing blasticidin sensitivity, and *BSD* and *VSG* 427-2 presence by PCR. As summarized in Figure 4 and Table 1, *VSG* GC and crossover rates increased ∼ 13- and ∼ 14-fold in the absence of RMI1, compared to wild type.

In our assay, switchers that arose by ES GC and ES loss cannot be distinguished, because these switchers will be negative for *BSD* and *VSG* 427-2 when examined by PCR [39]. Frequent loss of entire active ES was observed when *TK* was targeted next to the active ES promoter [45]. Therefore, *BSD^-^ VSG* 427-2^-^ switchers should represent either ES GC or ES loss (could be associated with multiple events). Deletion of *RMI1* decreased the rate of ‘ES GC or ES loss’, indicating that RMI1 is required for ES GC, a gene conversion event that could span ∼50 kb. Collectively, we conclude that RMI1 also controls the outcome of recombinational switching near *VSG*s, similarly to TOPO3α, and that RMI1 functions together with TOPO3α in *T. brucei* antigenic variation. Our data suggest that the RMI1-TOPO3α pathway has two distinct effects on *VSG* switching, suppressing recombination near the *VSG* but promoting recombination near the ES promoter.

### Genetic interaction between *TbRMI1* and *TbTOPO3α*

**Figure 5:**
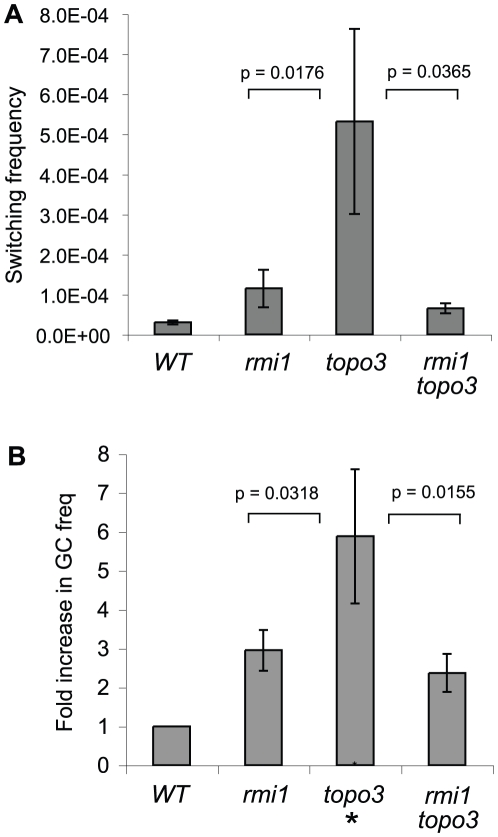
Genetic interaction between *T. brucei RMI1* and *TOPO3α*. (A) *TbRMI1* is epistatic to *TbTOPO3α* in *VSG* switching. (B) *RMI1* absence increases gene-conversion frequency at *URA3* locus and *TbRMI1* is epistatic to *TbTOPO3α* in gene conversion at this locus. Two counter-selectable markers, *TK* and *URA3*, were used to measure the GC rates [39]. *GCV^R^* and *FOA^R^* clones were counted and fold increase relative to wild type was plotted. Error bars indicate standard deviation. (*) in Figure 5B indicates *topo3α* data taken from the previous report [39]. Unpaired T tests were done and means of switching and GC frequencies were significantly different in the mutants.

Although TbRMI1 interacts with TbTOPO3α and the *rmi1* null mutation caused similar outcomes of *VSG* switching as the *topo3α*, RMI1 deficiency increased *VSG* switching frequency by only ∼ 4-fold, which is less than previously reported for TOPO3α deficiency [39]. If TOPO3α-RMI1 works in the same pathway, the *rmi1 topo3α* double mutant should exhibit the same phenotype as one of the single mutants. If *rmi1 topo3α* exhibits an additive phenotype, it is possible that they play additional separate roles. To determine the genetic relationship between RMI1 and TOPO3α, we compared the switching frequencies of wild type, *rmi1*, *topo3α^,^* and *rmi1 topo3α* double mutant. Switching was significantly increased in the *topo3α* single mutant (53.2±23.1×10^−5^), ∼ 10–30-fold higher than wild type (2.8±0.5×10^−5^), and this was ∼ 5-fold higher than *Tbrmi1* (11.0±3.4×10^−5^) (Figure 5A). The double mutant behaved more like *rmi1* single mutant, exhibiting only ∼ 2–3-fold increase (6.5±1.3×10^−5^) relative to wild type, suggesting that *RMI1* is epistatic to *TOPO3α* in antigenic switching. In the *rmi1 topo3α* double mutant, as summarized in Table 1, *VSG* switching also occurred mainly by *VSG* GC and crossover.

Next, we examined whether *Tbrmi1* causes hyper-recombination elsewhere and whether RMI1 is also epistatic to TOPO3α in this context. Previously, by replacing one allele of *TbURA3* with *HYG-TK*, we devised a recombination assay [39] in which GC at a non-ES locus can be measured using two counter-selecting drugs: GCV that kills *TK^+^* cells and 5-FOA that kills *URA3^+^*. The frequency of *GCV^R^* and *FOA^R^* represents the GC rate at the *URA3* locus. Using this assay, GC increased by 5.9-fold in the absence of TOPO3α [39]. One allele of *URA3* was replaced with *HYG-TK* in wild type, *rmi1* or *rmi1 topo3α* strains. Three independent *HYG^R^* clones each were examined and fold increase relative to wild type was plotted (Figure 5B). RMI1 deficiency increased GC rate (2.9-fold), and the double mutant behaved more like *rmi1* single mutant, exhibiting 2.4-fold increase of GC. The data show that *RMI1* is epistatic to *TOPO3α* in gene conversion at the*URA3* locus and in *VSG* switching.

### TbRMI1 is required for both RAD51-dependent and RAD51-independent *VSG* switching

**Figure 6:**
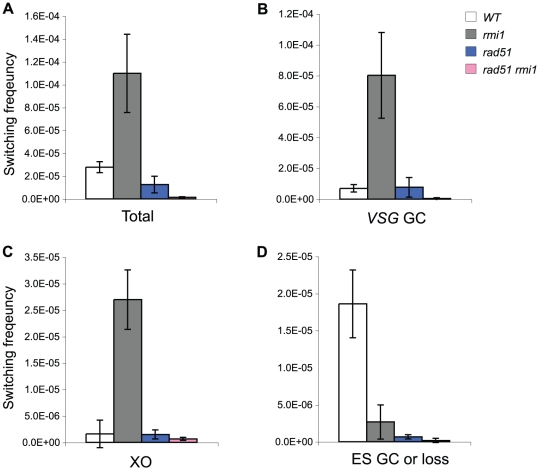
TbRMI1 is required for both RAD51-dependent and RAD51-independent *VSG* switching. (A) Simultaneous deletion of *RMI1* and *RAD51* severely impairs *VSG* switching. Overall *VSG* switching frequencies of wild type, *rmi1*, *rad51*, and *rmi1 rad51* were plotted. (B) Increased *VSG* GC rate in *rmi1* was dependent on RAD51, but RMI1 also functions in *VSG* GC switching independently of RAD51. (C) Increased crossover in *rmi1* was dependent on RAD51. (D) ‘ES GC or ES loss’ switching was impaired in a *rad51* or *rmi1* single mutant. The results are also summarized in Table 1.

The increased-switching phenotype of *Tbtopo3α* was dependent on *TbRAD51* [39]. Therefore, we asked whether RAD51 is required for the increased switching observed in the *rmi1* mutant. Both alleles of *RAD51* were deleted in wild type and *rmi1* mutant. *rad51* single and *rmi1 rad51* double mutants were examined for *VSG* switching frequency and mechanisms. 2×10^6^ cells from three independent cultures of each mutant were plated, and cloned *GCV^R^* switchers were analyzed. Deletion of *RAD51* caused only 2-fold reduction in overall *VSG*-switching in the presence of RMI1. On the other hand, it caused ∼ 19-fold reduction in the absence of RMI1, compared to wild type (Figure 6A and Table 1). This suggests that there are at least two recombination pathways working in antigenic switching mechanisms: *RAD51*-dependent and -independent.

75 and 10 cloned switchers were analyzed from *rad51* and *rmi1 rad51* mutants, respectively (Figure 6B, C, and D and Table 1). *VSG* GC was not affected in *rad51*, but it was significantly reduced in *rad51 rmi1* double mutants, indicating that at least two pathways are operating for *VSG* GC, one of which requires RMI1 independently of RAD51. *RAD51* deletion eliminated the increased *VSG* GC and crossover phenotype of *rmi1*, suggesting that RMI1 may be required to suppress RAD51-dependent recombinogenic structures arising between the active *VSG* and *VSG* donors, similar to the genetic interaction between *TOPO3α* and *RAD51*. Unlike *VSG* GC, ‘ES GC or ES loss’ was reduced in an *rmi1* or *rad51* single mutant, suggesting that RAD51-dependent recombinogenic structure may not be the cause of this switching mechanism. Therefore, recombination near ES promoter is fundamentally different from that near *VSG*. Collectively, we propose that RMI1 is required to suppress RAD51-dependent recombination intermediates near *VSG*, but it is required to promote recombination near ES promoter.

## Discussion

Extensive genetic and biochemical studies from yeasts have established the mechanisms of several key recombination pathways. These pathways serve multiple purposes, depending on cellular needs. Choice of recombination pathways determines cell fate. Failure to make the right choices can result in chromosome instability and/or in incompetence in responding to the environment, both eventually leading to high chance of lethality.

*T. brucei* expresses one type of *VSG* gene at a time, with the potential to express countless different *VSG*s. Switching to a different VSG allows parasites to escape a host's immune responses. As illustrated in Figure 3A, recombination-mediated *VSG* switching can be initiated near or downstream of the ES promoter. An entire silent ES or a relatively smaller region containing a silent *VSG* can be duplicated and transposed into the active ES to replace a previously active ES-associated *VSG*. This appears to occur through break-induced replication (BIR) [46], which is at least partly due to the 70-bp repeats that are present upstream of *VSG* genes, but limited homologies are present within and downstream of *VSG* genes. Telomere repeats may facilitate *VSG* switching, providing a second region of homology, but this possibility has not been extensively examined. How the recombination-mediated *VSG* switching occurs is still not clear.

Similar to mitotic recombination, crossover is relatively infrequent in recombination-mediated *VSG* switching [13]-[16], [39]. The RTR complex is one of the major factors that control mitotic crossover in other organisms. The RTR complex consists of a RecQ-family helicase (BLM/SGS1), a Topoisomerase III α, and RMI1/2 (BLAP75/18) [17]-[23]. *In vitro* studies showed that human hRMI1 stimulates the dHJ dissolution activity of TOPO3α and the dHJ unwinding activity of BLM-TOPO3α [35], [36].

**Figure 7:**
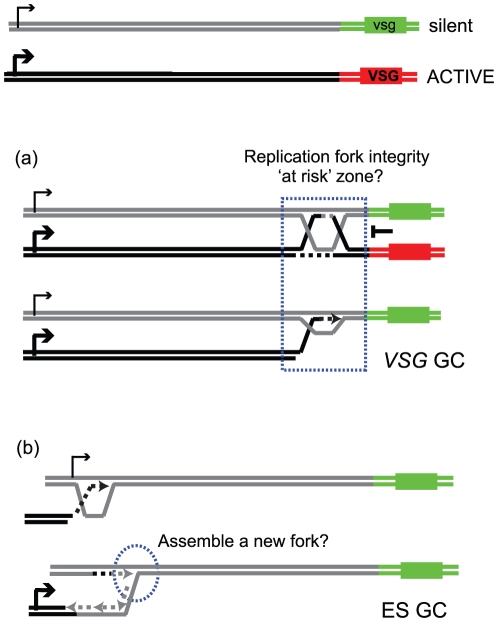
Multiple genetic networks in recombination-mediated *VSG* variation. The diagram shows the active ES (ES1) expressing *VSG* (red) and a silent ES containing *vsg* (green). RMI1 deficiency exhibited opposite phenotype in *VSG*-GC and ES GC switching, suggesting that molecular mechanisms of these events are distinct. We propose that there may be ‘replication fork instability zone(s)’ near *VSG*, potentially the 70-bp repeats, but not in ES promoter region. The RMI1-TOPO3α is required to dissolve recombination intermediates arising in this zone during replication. When their function is compromised, recombination intermediates accumulate and result in *VSG*-GC and crossover switches (a). RMI1 is required to promote ES GC. As ES GC requires extension of much longer regions compared to *VSG* GC, having a stable replication fork must be advantageous than having a migrating D-loop. Budding yeast SGS1 is required for replisome stability. Therefore, we propose that *T. brucei* RMI1-TOPO3α might stabilize a newly assembled replication fork between the active and a silent ES near promoter region. Formation of a stable replication fork may be the key step to generate ES GC switching (b).

We have previously shown that TOPO3α deficiency is associated with hyper-recombination, consistent with studies in other organisms, and with hyper-*VSG* switching phenotypes in *T. brucei* [39]. TOPO3α is required to suppress crossover *VSG* switching in *T. brucei*. In this study, we characterized a *T. brucei* RMI1 ortholog that physically interacts with TbTOPO3α. Similar to TOPO3α, deficiency of TbRMI1 increased the rate of *VSG* crossover, a hallmark of defects in RTR complex function. In addition, TbRMI1 deficiency increased *VSG* GC switching to a similar extent as TbTOPO3α deficiency, which caused dramatic increase in *VSG* GC rate. *VSG* GC was around 70% in both wild type and *Tbtopo3α* mutant, indicating that *VSG* GC frequency was 10-40 fold higher than wild type, as the overall *VSG* switching frequency was 10-40 fold higher in the *Tbtopo3α* mutant [39]. One of major functions of the RTR complex is to control recombination intermediates accumulating during replication stress. Genetic studies from budding yeast demonstrated that the growth defect of *top3* is caused by the accumulation of recombination intermediates and that the defect could be relieved by mutations in *SGS1* and in the *RAD51*-pathway [28], [32], [33]. Recombination intermediates accumulated when TOP3 enzyme activity was compromised [34]. Our data from this study suggests that RMI1 works together with TOPO3α during *VSG* switching. We propose that accumulation of recombination intermediates near the active *VSG* causes hyper-*VSG* GC and hyper-crossover phenotypes when TOPO3α-RMI1 function is compromised (Figure 7 and [39]).

What could cause recombination intermediates to accumulate in the active *VSG* locus? Genome-wide analysis of ORC1 association showed that ORC1 was enriched at subtelomeric regions (Kinetoplastid Molecular Cell Biology meeting April 2011, Tiengwe CM et al.). Some of these ORC1 binding sites may initiate replication. *VSG*s are located close to telomere repeats. It was shown that the telomere repeats are fragile in mammalian cells, dependent on replication and telomere binding factor TRF1 [47]. In the absence of TRF1, telomere repeats break more frequently and replication was frequently initiated within the TTAGGG repeats, increasing telomere instability and sister-chromatid bridging. Interestingly, BLM and RTEL helicases were required to suppress the telomere fragility. Thus, it is possible that fragility of the active ES could be due to a combination of telomere instability and replication defects. In the absence of TOPO3α or RMI1, these could cause the accumulation of recombination intermediates.

Due to lack of information on DNA replication in *T. brucei*, it is impossible to determine the identities of recombination intermediates that might accumulate in *topo3α* or *rmi1* trypanosome mutants. Recombination intermediates could arise when replication forks encounter DNA damage, during replication progression and repair. Intermediates could form between sister-chromatids with entangled regions, called hemicatenanes, which can be processed to ‘rec-X’ structures, whose resolution requires SGS1-TOP3 in budding yeast [34], [48], [49]. These structures are different in regard to base pairing and presence of single stranded regions and they can be visualized by DNA 2D gel analysis by Southern blot, a technique unavailable in *T. brucei*. We showed that *VSG* GC and crossover are suppressed by TOPO3α and RMI1 in a RAD51-dependent manner. Therefore, we think that TOPO3α-RMI1 may play roles in maintaining ES integrity near *VSG*s by removing these ‘unwanted’ recombination intermediates. Without TOPO3α-RMI1, they might be resolved by other nucleases (crossover), or a silent *VSG* locus might be used as a new template during hemicatenane-mediated template switching using its homology sequences and complete replication (*VSG* GC). It is possible that TOPO3α-RMI1 deficiency could be associated with location specific *VSG* switching phenotypes due to replication stability ‘at risk’ zone(s) near *VSG* genes. In the normal situation, TOPO3α-RMI1 may be responsible for protecting the integrity of that region(s) (Figure 7(a)).

When double strand breaks (DSBs) occur, broken DNA ends are resected, leaving a 3′ overhang, and a RAD51 filament on single stranded DNA invades a template strand to form a D-loop. How this structure is processed can decide how the breaks are repaired. End resection requires SGS1-DNA2-RPA, and EXO1, and SAE2-MRE11 complex and the end resection activity of SGS1 is stimulated by TOP3-RMI1 in budding yeast [50], [51]. BIR frequency increases 39-fold in *sgs1Δ exo1Δ* double mutants [52]. Interestingly, in combination with *exo1Δ*, BIR was increased in a separation-of-function allele of *SGS1*, *sgs1-D664Δ*, which is proficient for gene conversion but defective for resolution of recombinogenic X-structures formed by replication fork stalling [52], [53]. The residue D664 is located at the second acidic region (AR2) in the N-terminus of SGS1 and the *sgs1-AR2Δ* shows similar phenotype as the *sgs1-D664Δ*. There are two recQ family helicases in *T. brucei*, Tb927.8.6690 and Tb927.6.3580, and Tb927.8.6690 appears to be a BLM/SGS1 ortholog. The region encompassing D664 of SGS1 seems to be conserved in Tb927.8.6690; YPWS**D**_664_E in SGS1 and YPWS**E**_449_E in Tb927.8.6690. Tb927.8.6690 and mutations at this particular region would be useful tools to study molecular mechanism of *VSG* switching more in detail in the future.

It is notable that *VSG* GC frequency decreased further in *rmi1 rad51* double mutants compared to wild type, suggesting that there are pathways that require *RMI1*, independently of *RAD51*. Studies in yeasts and in *T. brucei* have shown that recombination still occurs in *rad51Δ* cells [54]-[56]. In the absence of telomerase, telomeres are maintained by a RAD51-dependent and independent pathways [57]-[59]. It is possible that RMI1 may play distinct roles in *VSG* switching in a RAD51-independent manner.

The frequency of ‘ES GC or ES loss’ decreased in the absence of RMI1 or RAD51, unlike *VSG* GC, which was significantly increased in the absence of RMI1 and was not affected by RAD51. This is interesting but puzzling, considering that hyper-recombination is the hallmark phenotype of RMI1 deficiency. ‘ES GC or ES loss’ switching seems to occur in a RAD51 and/or RMI1 dependent manner, but their genetic relations cannot be deduced from the current data. However, it is clear that ES GC is not triggered by the same source as the *VSG* GC. Random breaks may occur at the active promoter due to high level of transcription and these breaks would normally be repaired by homologous recombination, using sister-chromatids, or by NHEJ. However, infrequently, template switching (to a silent ES promoter region) could occur and be repaired by BIR to generate ES GC switchers. When BIR was assayed in a budding yeast cell line with two template chromosomes available, template switching occurred about 20 percent of the time, usually near the sites of strand invasion [60]. The current hypothesis is that repair could be completed by D-loop migration coupled with lagging strand DNA synthesis moving along the template or strand invasion intermediates can be cleaved by endonuclease(s) [60], [61]. This cleavage would allow a new replication fork to establish between two chromosomes (the active and a silent ES in the case of *VSG* switching). As ES GC switching requires duplication of much larger fragment (∼ 50 kb) than the *VSG* GC (∼ 4 kb), establishing a stable replication fork could be more advantageous than a migrating D-loop for ES GC, while it would not matter for *VSG* GC events that require duplication of shorter regions. The repair can be completed by a newly assembled replication fork between the active and silent ES, giving rise to an ES GC switcher (Figure 7 (b)).

In yeast, SGS1 is required for replisome stability, affecting the assembly of replication factors at the fork, in collaboration with checkpoint kinase MEC1 [62]. Therefore, TbRMI1-TOPO3α might be required for the stability of a newly formed replication fork between the active and a silent ES promoter region. If this is the case, generation of ES GC switching should require RAD51 and RMI1-TOPO3α functions. Alternatively, RMI1 might have novel function in the context of ES promoter, which may indirectly affect RAD51-dependent BIR. Transcription of ES occurs polycistronically with a *VSG* located ∼ 50 kb downstream of the promoter, the only site where transcription machinery assembles within the unit. ES GC and *VSG* GC occur in different chromatin environments, so the mechanisms of these gene conversions could be fundamentally distinct.

The *rmi1* mutant was epistatic to *topo3α* in *VSG* switching phenotype. This genetic interaction is interesting because one would expect the opposite scenario considering that TOPO3α possesses enzymatic activity for resolution of recombination intermediates, which is stimulated by RMI1 [35], [36]. The epistatic interaction of TbRMI1 and TbTOPO3α in *VSG* switching suggests that TbRMI1 may have a role, potentially with BLM helicase, in a process upstream of TbTOPO3α.

Given high level of sequence similarities between ESs, it is likely that silent ESs can recombine at a certain rate and yet the ES structures seem to be well maintained, suggesting that silent ES recombination may also be suppressed by protectors of genome integrity, and recombination of silent ESs might be elevated in *topo3α* or *rmi1* trypanosome mutants. It is difficult to examine recombination rates at or between silent ESs quantitatively, due to technical difficulties, such as strong gene silencing and sequence similarities.

It appears that trypanosomes suppress *VSG* GC effectively, although *VSG* switching is essential for parasite survival in the host and *VSG* GC is a dominant immune evasion mechanism. During host infection, parasite numbers rise and fall periodically. We think that recombination-mediated *VSG* switching is generally suppressed but one or two silent *VSG* loci may have a higher chance to recombine with the active ES, potentially due to their proximity. *VSG* loci might be highly recombinogenic without this suppression, which could increase the possibility for parasites to use up their expressible *VSG*s at the early stage of infection, consequently resulting in eradication by host immunity. To prevent this, parasites may expose their ES-linked *VSG* repertoire gradually to preserve the population survival by *VSG* switching and to prolong their infection, while having some time to accumulate novel *VSG*s at transcribable loci.

*VSG* switching requires key factors in recombination and replication and their interplay controls the outcome of *VSG* switches. Although it is beginning to reveal its mystery, the control mechanism of antigenic switching must involve various cellular processes, whose contributions are largely unknown. Additional factors as well as specific alleles of the RTR complex members should be studied thoroughly to understand mechanism of antigenic variation in depth.

## Materials and Methods

### Trypanosome strains and plasmids

*Trypanosoma brucei* bloodstream forms (strain Lister 427 antigenic type MITat1.2 clone 221a (expressing *VSG* 427-2)) were cultured in HMI-9 at 37°C. The cell lines constructed for this study are listed in Table 2. They are of ‘single marker (SM)’ background that expresses T7 RNA polymerase and Tet repressor [63]. Stable clones were obtained and maintained in HMI-9 media containing necessary antibiotics at the following concentrations, unless otherwise stated: 2.5 µg/ml, G418 (Sigma); 5 µg/ml, blasticidin (Invivogen); 5 µg/ml, hygromycin (Sigma); 0.1 µg/ml, puromycin (Sigma); 1 µg/ml, phleomycin (Invivogen). Plasmids used for this study are listed in Table 3.

**Table 2:** Strains used in this study.

Names (sources)	Genotypes	Manipulations
*SM*	*WT*	*T7 RNA polymerase* and *Tet repressor (TetR):: NEO*
*HSTB-188* [39]	*WT*	*ES1 promoter::BSD*
*HSTB-261* [39]	*WT*	*ES1 promoter::BSD, 70-bp repeats-PUR-TK*
*HSTB-229*	*rmi1^-/+^*	*ES1 promoter::BSD, rmi1ΔloxP-HYG-TK-loxP/+*
*HSTB-286*	*rmi1^-/-^*	*ES1 promoter::BSD, rmi1ΔloxP-HYG-TK-loxP/ΔloxP-PUR-loxP*
*HSTB-298*	*rmi1^-/-^*	*ES1 promoter::BSD, rmi1ΔloxP/ΔloxP*
*HSTB-336*	*rmi1^-/-^*	*ES1 promoter::BSD, 70-bp repeats-PUR-TK, rmi1ΔloxP/ΔloxP*
*HSTB-411, 412, 413*	*rmi1^-/-^ + Vector*	*ES1 promoter::BSD, 70-bp repeats-PUR-TK, rmi1ΔloxP/ΔloxP* *+ pHD309 (empty vector)*
*HSTB-414, 415, 416*	*rmi1^-/-^ + RMI1-wt*	*ES1 promoter::BSD, 70-bp repeats-PUR-TK, rmi1ΔloxP/ΔloxP* *+ pSY38 (RMI1-wt in pHD309)*
*HSTB-344* [39]	*topo3α^-/-^*	*ES1 promoter::BSD, 70-bp repeats-PUR-TK, topo3αΔloxP/ΔloxP*
*HSTB-365* [39]	*rad51^-/-^*	*ES1 promoter::BSD, 70-bp repeats-PUR-TK, rad51ΔHYG/rad51ΔPHELO*
*HSTB-384, 385, 386*	*rmi1^-/-^ rad51^-/-^*	*ES1 promoter::BSD, 70-bp repeats-PUR-TK, rmi1ΔloxP/ΔloxP,* *rad51ΔHYG/rad51ΔPHELO*
*HSTB-456*	*rmi1^-/-^ topo3α^-/-^*	*ES1 promoter::BSD, 70-bp repeats-PUR-TK, rmi1ΔloxP/ΔloxP, topo3αΔloxP/ΔloxP*
*HSTB-201*	*2040-3HA* (*)	*2040-3HA::HYG*
*HSTB-210*	*RMI1-3HA*	*RMI1-3HA::PUR*
*HSTB-213*	*TOPO3α-3MYC*	*TOPO3α-3MYC::PHLEO*
*HSTB-218*	*2040-3HA(* * *) TOPO3α-3MYC*	*2040-3HA::HYG, TOPO3α-3MYC::PHLEO*
*HSTB-222*	*RMI1-3HA TOPO3α-3MYC*	*RMI1-3HA::PUR, TOPO3α-3MYC::PHLEO*

(*) The full annotation is Tb927.8.2040.

**Table 3:** Plasmids used in this study.

Names	Inserts, targeting loci, and markers	Sources
pHD309	Vector to target at *TUB* array, *HYG*	
pLHTL-pyrFE	*TbURA3::HYG-TK*	[64]
pLEW100-Cre	Cre-recombinase in expression vector, *PHLEO*	[64]
pHJ17	*loxP-HYG-TK-loxP*	[39]
pHJ18	*loxP-PUR-TK-loxP*	[39]
pHJ23	To target *BSD* downstream of ES promoter	[39]
pHJ63	*TOPO3α Δ::loxP-PUR-TK-loxP*	[39]
pHJ64	*TOPO3α Δ::loxP-HYG-TK-loxP*	[39]
pSY1	*RAD51Δ::HYG*	[39]
pSY23	*RAD51Δ::PHLEO*	[39]
pHJ71	*RMI1Δ::loxP-HYG-TK-loxP*	This study
pHJ77	*RMI1Δ:: loxP-PUR-loxP*	This study
pSY38	To insert wild-type *RMI1* at *TUB* array, *HYG*	This study
pMOTag53M	One-step PCR-3xMYC tagging construct, *PHLEO*	[42]
pMOTag4H	One-step PCR-3xHA tagging construct, *HYG*	[42]
pMOTag2H	One-step PCR-3xHA tagging construct, *PUR*	[42]

### Construction of *Tbrmi1* knock-out lines

To generate *T. brucei rmi1* mutants, HSTB-188, a wild-type ‘single marker’ line with the active ES marked with a blasticidin-resistance gene (*BSD*), was sequentially transfected with deletion cassettes. The cassettes contain either a hygromycin-resistance gene conjugated with a *Herpes simplex* virus thymidine kinase (*HYG-TK*) or a puromycin-resistance gene (*PUR*) flanked by homology sequences to upstream and downstream of Tb927.3.1830. Deletions were confirmed by PCR analyses. Single knock-out (sKO), *rmi1^-/+^*, and double KO, *rmi1^-/-^* were used to assess growth phenotypes, as described previously [39]. Selection markers flanked by loxP sites were removed by transiently expressing Cre-recombinase (pLew100-Cre). The cells that lost both *HYG-TK* and *PUR* were selected in 50 µg/ml ganciclovir (GCV). Loss of markers was confirmed by resistance to puromycin and hygromycin, and by PCR analysis. The sequences of primers used here are available upon request. These cell lines were used for recombination and *VSG* switching assays.

### Co-immunoprecipitation and western blot

About 10^8^ cells were lysed in lysis buffer (25 mM Tris–HCl (pH 7.5), 1 mM EDTA, 0.5% NP-40, 10% glycerol, 1 mM phenylmethylsulfonylfloride (PMSF), 1 mM dithiothreitol (DTT), protease inhibitor cocktails (Sigma)). Whole cell lysates were immunoprecipitated with rabbit anti-HA or anti-MYC antibodies. Immunoprecipitates were analyzed by western blot using mouse anti-HA or anti-MYC antibodies.

### *VSG* switching assay and analyses of switchers

To create switching reporter strains, the promoter and 70-bp repeats of the active ES were marked with *BSD* and *PUR-TK*, respectively, as described previously [39]. To determine *VSG* switching frequency and mechanisms, cells were maintained in the presence of blasticidin and puromycin to exclude switchers from the starting population. Cells were then allowed to switch in the absence of drug selection for 3 days. Indicated numbers of cells (Table 1) were suspended in HMI-9 media containing 5–10 µg/ml GCV and distributed in 96-well plates. GCV-resistant clones were examined for sensitivity to 2 µg/ml puromycin, to exclude non-switchers that carry spontaneous mutation(s) in *TK* but not in *PUR* gene. Non-switchers that carry mutations both in *PUR* and *TK* were ruled out by western blot analysis using anti-VSG 427-2 antibodies. Cloned switchers were analyzed for blasticidin sensitivity. Genomic DNA was analyzed by PCR for the presence of *BSD* and *VSG* 427-2. The sequences of primers used here are available upon request.

It is noted that all *VSG* switching assays in this study were performed without the MACS-enrichment step. MACS step may have reduced the number of some switchers, as ‘ES GC or ES loss’ switchers appear to have growth disadvantages, compared to parental cells and *VSG* GC switchers.

### Recombination assay

Gene-conversion frequency at *TbURA3* locus was determined as described previously [39].
